# The Enhanced Games: a timely injection for the International Olympic Committee

**DOI:** 10.3389/fspor.2024.1490651

**Published:** 2024-10-16

**Authors:** David Ekdahl, Jörg Krieger

**Affiliations:** ^1^ Department of Public Health, Faculty of Health, Aarhus University, Aarhus, Denmark; ^2^ Lillehammer Olympic and Paralympic Study Center, Innlandet Business School, Inland Norway University of Applied Sciences, Elverum, Norway

**Keywords:** IOC, Enhanced Games, sustainability, sports governance, performance-enhancing drugs, Olympic Games, megaevents, athlete-centered

## Abstract

The Enhanced Games, a privately funded sporting megaevent aspiring to rival the Olympic Games, have garnered significant media attention since its public inception in 2023. This attention has primarily been driven by the Enhanced Games’ embrace of performance-enhancing drugs. Lost in the public fixation on the event’s green-lit drug-use, however, is the fact that the Enhanced Games distance themselves from current Olympic standards in numerous ways beyond drug-policies alone. More precisely, the Enhanced Games promote themselves as a more economically and ecologically sustainable alternative to the Olympics, as well as a megaevent that aims to put athletes and their safety front-and-center. With an eye towards current Olympic standards, we suggest that closer examination of the Enhanced Games offers novel perspectives on the future of the Olympics and global sporting events more broadly.

## Introduction

The Enhanced Games (TEG) are an attempt to establish an alternative sporting event to traditional large-scale sporting formats – especially the Olympic Games. TEG present themselves as a fundamentally futuristic venture, aiming to replace the sporting ideals and traditions of an allegedly outdated Olympics with a new, techno-scientifically infused megaevent. With a transhumanistic ambition to fully “embrace science and technology”, the people behind TEG established the project to “push the boundaries of human performance”, especially through the greenlighting of athletes’ use of performance-enhancing drugs (PEDs) (1).

The media- and academic interest has concentrated on TEG's sanctioning of PEDs, too (2–6). At the 2024 Conference of the International Network of Doping Research, TEG founder Aron D'Souza discussed and defended the enhancement procedures for athletes’ participating at TEG (7). During the conference, a clearer picture of TEG as more than a PED-infused mirroring of the Olympic Games emerged. More precisely, TEG's sales pitch, fleshed out in large part as a critical response to the Olympic Games, revolves not only around the controversial topic of PED accessibility, but also, in theory, around ambitions of athlete-focus and inclusivity, as well as of ecological and economical sustainability.

Before moving on, we stress that this perspective piece is not an endorsement of the Enhanced Games. As sports researchers, we remain highly sceptical of the overall project. Our primary aim here is to *disentangle* TEG, identifying and analysing the distinct features of the proposed project. Our reasons for this disentanglement are threefold. First, as a proposed, complete alternative to the Olympic Games, TEG as a project is in fact a *bundle* of organisational ideas. Without disentanglement of the controversial from the serviceable features, TEG will continue to package valuable reproaches of the IOC together with potentially harmful ideas. Second, without unscrambling the valuable from the harmful features of TEG, the IOC are in a position to potentially distance themselves from all of these features as precisely a single package of ideas. This is especially applicable should TEG fail as a project, potentially allowing the IOC to point at this hypothetical failure as justification for dismissing all of TEG's distinct suggestions. Critically assessing the distinct features of TEG, both the serviceable and the potentially harmful, means treating them as potential lessons that might, at the very least, allow for fresh perspectives on the future of large-scale sporting events like the Olympics. Finally, we consider fostering dialogue between what might be perceived as entirely opposing ideas as a core task of the academic community. It is evident that the Olympic Movement, despite the – on initial inspection – success of the 2024 Paris Olympic Games, faces considerable future challenges (8). A turn towards unconventional ideas might contribute to finding solutions to such challenges. We thus regard this piece as a prompt for the IOC to come out of its echo chamber and engage with alternative perspectives, such as those proposed by TEG.

## Sustainability

Cities often go over budget when hosting the Olympics. It has gotten to the point where the fact that the 2024 Olympic Games in Paris came in 25% over the initial budget, with total spending just shy of $10 billion, is treated as newsworthy and a potential source of inspiration for future Olympic Games by major news outlets (9–14). For comparison, some estimates put budget deviations for the 2016 Olympic Games in Rio at over 350% (9), although the precise deviations are contested (15).

Hosting the Olympic Games have often had considerable, negative impact on host cities. One of the more infamous cases is the 1976 Summer Olympics of Montreal, which placed such considerable debt on the Canadian city, it took over 30 years to repay it (16). A more recent example is the 2016 Olympics in Rio, which caused the city to default as a result of debt load, going into its gravest recession in close to a century (17). At the heart of such Olympic budget deviations, amongst other things, are the infrastructure projects associated with hosting the Olympics, which were a particularly high source of expenses for a city like Rio to construct and maintain (18).

Around the turn of 2015 the IOC released their “Olympic Agenda 2020” (19), with the clearly stated goal of increasing the manageability of hosting the megaevent for the host cities. A core feature of the agenda was an increased flexibility on the side of the host cities to rely on existing infrastructure and “the use of temporary and demountable venues where no long-term venue legacy need exists or can be justified” (19, 20).

While the ambition of reducing cost, in part by prioritising host cities with existing infrastructure, is laudable from a waste-reduction perspective, its concrete impact remains uncertain. In addition, an obvious worry is that this prioritising makes for an uneven playing field between cities, and risks shutting out of the bid cities that cannot sustainably foot the total costs associated with the Olympic Games.

Several suggestions have been raised for mitigating the risk of budget deviations (21–24), with the most immediately relevant ones for our purposes relating to the current Olympic city hosting logic. In this regard, some propose letting the same city host the Olympic Games several times in a row (25). Other researchers, favoring an even more centralized and stable model, suggest that the Summer Olympics and the Winter Olympics both be hosted at permanently set locations, rather than fluctuating between new host cities each year or every other year (22).

One problem with an overly centralized hosting model is the further loss of the “global village”-appeal of the Olympics: the bringing together not just of athletes and audiences around the world, but an anchoring of this in different cities across the world in a celebration of global, cultural diversity.

In this context, echoing the above skepticism towards the current system, TEG advocate a model of increased de-centralization; one of spreading out their event across the world, rather than picking a single host city to carry the financial burden of the megaevent (cf. 24).

We suspect the spectacle produced by such a hosting structure, as compared to a more stable and centralized model, resonates with the ideals of the Olympics, putting before the global audience an ever-changing network of cities spread across the world, collaborating in their roles as Olympic hosts. On this model, much of the cost of hosting would be made significantly more cost-efficient; the distinct pieces of infrastructure needed more readily available on a wider, global scale. Moreover, such a model notably provides a more flexible bidding scheme and could more readily carry standards of geographic inclusivity.

Inseparable from the economic challenges following the current Olympic hosting logic is also its obvious environmental impact. Not only is the Olympics, in lieu of the megaevent's sheer magnitude (and potential place as a role model for other large-scale events), a significant factor when it comes to global carbon emissions, it is also, conversely, in danger itself of becoming non-hostable in its current format in up to 27% of cities around the world by the end of the twenty-first century as an immediate result of global warming (26).

Several proposals have been raised to increase the climate viability of the Olympics, such as downsizing the event, as well as increasing sustainability governance and thus responsibility for hosts that fail to meet set climate goals (27). Given that the environmental impact of the Olympics is in large part driven by the construction of new infrastructure, as well as the carbon footprint of tourists traveling from all over the world to the host city (27), a more de-centralized hosting model like that of TEG may well be an effective way of improving the ecological sustainability of the megaevent. For one, since infrastructure requirements would be distributed across host cities, the need to construct infrastructure locally could be significantly scaled back. Moreover, the overall travel-time and cost to participate in the Olympics would be reduced, especially if the games were hosted with a more evenly, e.g., continent-based, host city distribution. In this regard, a de-centralized model would further increase the global accessibility of the Olympics for nations around the world, providing not just a more sustainable but also a more globally inclusive megaevent.

## Athlete-centrism

Scholars, athletes’ groups, and the media have in recent years voiced considerable criticism of the IOC's approach towards the positioning of athletes within the structures of the global sport system (28–31). Evidently, TEG are aware of this criticism and have adopted a position in public communication that highlights the apparent inequalities of the Olympic system (32). Athlete-centrism is a key part of TEG's marketing strategy as the needs, rights, and achievements of athletes are centrally promoted. In doing so, TEG build on the public criticism of traditional institutional control mechanisms within the Olympic Movement and instead promote individual, athlete-centred empowerment. Since criticism of the IOC's handling of athletes has centred around issues of national representation and financial benefits, we focus on these two elements.

With the exception of the Olympic Refugee Team, all athletes participating in the Olympic Games must represent a nation and be registered through a National Olympic Committee (NOC). This fundamental principle of national representation has been a core aspect of the Olympic Movement since its establishment at the end of the nineteenth century. The Olympics’ founder Pierre de Coubertin envisioned his sport event as a means to promote peace amongst nations and integrated national symbols such as flags and anthems into the ceremonial parts of the Games (33).

Several developments in recent decades have challenged the traditional sport organizations’ adherence to the nation-concept. In particular, globalization and migration processes have redefined senses of belonging and created global citizens with multiple identities. As a result, athletes are switching citizenships and national affiliations more regularly, some competing for different nations at different Olympic Games. Rich states are attracting talented athletes from all over the world to represent their colours in exchange for monetary benefits (34). Moreover, athletes might not necessarily identify with one single nation only, instead adopting a more fluid national identity. Today, sport is an arena in which national ties have become of decreasing importance and the necessity for a more flexible approach towards nationality (35).

TEG's focus on the rights and liberties of individual athletes addresses this problem directly. Athletes competing at TEG events will not represent a nation that might instrumentalize an individual's success. Rather, TEG argue, their approach ensures that individual athletes’ rights are not sacrificed for national interests. This model is grounded in a civil-rights framework that is prominently cited in TEG's public communication (36). In doing so, TEG provide a platform for the promotion of liquid national identities in elite sport; a concept that one of the authors has recently explored and discussed extensively (37, 38).

In addition, to this day, athletes do not receive any prize money or direct financial benefits of the revenues generated through the Olympic Games. This policy is rooted in the amateur history of the Olympic Movement (39). Such restrictions come on top of regulations in the Olympic Charter that prevent athletes from showing their personal sponsors’ logos or other individual brand associations. Rule 40 of the Olympic Charter had established for decades that participants at the Olympic Games were not permitted to connect their sporting performance with personal advertising campaigns during the time of the event. The rule's primary purpose is to safeguard the exclusive rights of official Olympic sponsors on the Olympics’ intellectual property, thus protecting the IOC's revenues through commercial partnerships (40).

Leading up to the 2020 Tokyo Olympic Games, the rule was loosened, and athletes have been provided with more latitude and more commercial opportunities (41). Non-Olympic, personal sponsors can now advertise successes of their contracted athletes in connection with the Olympic Games, and athletes can post “thank you” messages on their social media accounts. However, various requirements and rules remain in place. For example, athletes cannot mention their sponsors at official press conferences nor within the sporting locations (41).

Since Olympic athletes represent nations, their financial rewards are arranged on a national level with vast differences across countries and sports (42). With TEG's focus on individual athletes without national representation, it is evident that an alternative system is required. TEG founder D'Souza is well aware of the lack of central support for Olympic athletes: “The underpayment of athletes is the core moral failing of the Olympic movement — it is a vestige of the aristocratic sentiment behind the outdated unpaid ‘amateur’ requirement” (43). In this spirit, TEG provide leverage for all participating athletes as it promises six-figure base salaries. In addition, there is prize money of $1 million for each achieved world record.

There are stark differences in participation numbers to consider. More than 10,000 athletes competed at the 2024 Olympic Games, whereas TEG plan to involve less than 100 athletes. However, it appears that some sport federations have reacted to the new financial incentives promised by TEG. In April 2024, World Athletics, the governing body of track and field, decided to award gold medal winners at the Olympic Games with a cash prize of $50,000 (€46.7k). This decision represents a significant departure from IOC policy. TEG notably claim that their plans impacted the World Athletics’ decision (44). Whilst this is difficult to assess, at the very least, TEG's financial model has arguably triggered reconsiderations of the flawed financial model within the Olympic system.

## Safety

Whilst the use of PEDs has long been associated with increased health risks, PED regulation is not without its own inherent risks either (45, 46). As emphasised by Henning et al. (47), PED regulation carries with it a broader “risk environment”, ostensibly minimizing the risks of physical harm to the athletes at the cost of enhancing their “social, economic and political risks” in large part through stigmatization of PED-use (48, 49). A result of such stigmatization is precisely an environment that incentivizes PED-use opaqueness and with potentially increased risks (47).

In addition, the current anti-doping regime controlled by the World Anti-Doping Agency (WADA) is not without failures either. As evidenced by the recent scandal around 23 Chinese swimmers, WADA allegedly mishandled the case and showed severe governance failures (50).

TEG tie a political dimension to the stigmatization of PED-use, colouring it in language of political inclusivity and equality (51). In this regard, with its stated ambition of de-stigmatization, TEG seemingly aim to circumvent some of the more systematic, social risks inherent in current anti-doping efforts, including in the Olympic Games. TEG also commented on the Chinese case and highlighted that its own approach to drug use in sport would enhance sporting integrity (52).

TEG advocate for this particular brand of athletic liberation whilst maintaining an ideal of ensuring athletes’ physical safety even within a PED-greenlit context. While the practical and moral plausibility of this is arguably doubtful, TEG's concrete strategies for protecting athletes’ physical wellbeing in PED contexts are particularly interesting compared to current IOC-standards. For example, TEG do not allow *all* substances to be used in competition, and drugs are only allowed to be taken under medical supervision to prevent overuse or risks due to inherited health issues (44). Thus, TEG athletes’ health statuses are constantly evaluated, a recommendation that leading international doping scientists made to the IOC already fifteen years ago (53).

## Concluding remarks

In our opinion, there are good medical, philosophical (especially ethical) as well as practical reasons to remain highly sceptical of TEG. Some of these include the inherent physical risks associated with PED use, the uncertainty vis-à-vis actual athlete recruitment, and the possible biotechnical transformation of sports’ telos altogether stemming in large part from TEG's transhumanistic ideals. Moreover, a fundamental tension persists between the project's liberal aspirations towards individual athletic “freedom to enhance” and its seemingly paternalistic need to still regulate PED use close enough to label the event as safe and supervised. Given TEG's ambition of functioning effectively as sports-based research grounds for technology- and science-driven human enhancement (54), we also find the opaqueness and potential for misuse associated with the collection and storage of various biomarkers from participating athletes to be troublesome. It is, finally, important to keep in mind that TEG plans are still not fully fledged out and remain of a theoretical nature. A start date for the second half of 2025 has been announced, but no evidence for its realization has been provided.

Nevertheless, key ambitions of TEG suggest that the movement is in some areas more advanced and in touch with reality than current Olympic standards, as well as standards of traditional sport organizations more broadly. Our recommendations are that the IOC, and relevant sporting organizations, consider TEG's novel suggestions for sustainability, athlete representation, payments and inclusion, as well as the rhetoric around harm reduction as central and distinct points of interest moving forward.

## Data Availability

The original contributions presented in the study are included in the article/Supplementary Material, further inquiries can be directed to the corresponding author.
